# Development of multiplex PCR and multi-color fluorescent in situ hybridization (*m*-FISH) coupled protocol for detection and imaging of multi-pathogens involved in inflammatory bowel disease

**DOI:** 10.1186/s13099-018-0278-1

**Published:** 2018-12-05

**Authors:** Robert C. Sharp, Ebraheem S. Naser, Karel P. Alcedo, Ahmad Qasem, Latifa S. Abdelli, Saleh A. Naser

**Affiliations:** Division of Molecular Microbiology, Burnett School of Biomedical Sciences, University of Central Florida College of Medicine, 4110 Libra Drive, Orlando, FL USA

**Keywords:** Crohn’s disease, Ulcerative colitis, MAP, *m*-FISH, Multiplex PCR

## Abstract

**Background:**

Several pathogens have been debated to play a role in inflammatory bowel disease (IBD) including Crohn’s disease (CD). None of these pathogens have been investigated together in same clinical samples. We developed a multiplex PCR and multi-color fluorescent in situ hybridization (*m*-FISH) protocols for simultaneous detection of CD-associated pathogens including *Mycobacterium avium* subspecies *paratuberculosis* (MAP), *Klebsiella pneumoniae*, and adherent-invasive *Escherichia coli* strain LF82.

**Methods:**

The multiplex PCR is based on 1-h DNAzol^®^ extraction protocol modified for rapid extraction of bacterial DNA from culture, blood, and intestinal biopsies. Oligonucleotide primers sequences unique to these pathogens were evaluated individually and in combinations using bioinformatics and experimental approaches. *m*-FISH was based on fluorescent-tagged oligonucleotides and confocal scanning laser microscopy (CSLM).

**Results:**

Following several attempts, the concentration of the oligonucleotide primers and DNA templates and the PCR annealing temperatures were optimized. Multiplex PCR analyses revealed excellent amplification signal in trials where a single primer set and combinations of two and three primers sets were tested against a mixture of DNA from three different bacteria or a mixture of three bacterial cultures mixed in one tube before DNA extraction. Slides with individual and mixtures of bacterial cultures and intestinal tissue sections from IBD patients were tested by *m*-FISH and the CSLM images verified multiplex PCR results detected on 3% agarose gel.

**Conclusion:**

We developed a 4-h multiplex PCR protocol, which was validated by *m*-FISH images, capable of detecting up to four genes from major pathogens associated with CD. The new protocol should serve as an excellent tool to support efforts to study multi-pathogens involved in CD and other autoimmune disease.

## Background

Inflammatory bowel disease (IBD), which consists of Crohn’s disease (CD) and ulcerative colitis (UC), share a variety of different genetic factors, environmental triggers, and treatment plans [1–5]. Multiple recurring reports from our laboratory and others provided evidence that environmental triggers including enteric pathogens may be associated with IBD. Specifically *Mycobacterium avium* subspecies *paratuberculosis* (MAP), adherent-invasive *Escherichia coli* (AIEC) strain LF82, and *Klebsiella pneumoniae* have been implicated as possible causative agents in CD [5–12].

MAP was first isolated as the causative agent for Johne’s disease, a CD-like enteritis in cattle [8, 9, 13, 14]. MAP is an intracellular acid-fast pathogen that infects macrophages and dendritic cells and inhibits phagosome–lysosome fusion [15, 16]. Interestingly, MAP was detected in the blood, milk and intestinal biopsies from patients with CD and most recently from the blood of Rheumatoid Arthritis (RA) [5, 6, 8, 9]. On the other hand, AIEC is a gram-negative bacillus pathogen that has also been isolated from intestinal tissue from patients with CD [10, 12, 17, 18]. AIEC strain LF82 has been studied intensely due to its ability to infiltrate intestinal tissue and increase pro-inflammatory cytokines level in CD patients [10, 12, 17, 18]. Like MAP, AIEC strain LF82 resists phagosome–lysosome fusion and acidification [10, 12, 17, 18]. Recent studies also showed that *K. pneumoniae* might be associated with IBD pathogenesis [11, 19]. *K. pneumoniae* is a gram-negative, facultative anaerobic bacillus pathogen that causes pneumonia in immunocompromised patients [11, 19]. It colonizes the intestinal epithelial with an imbalance in gut flora leading to elevated humoral immune response [11, 19].

Although there are numerous studies investigating the role of these pathogens in CD pathogenesis, none of the studies examined the co-occurrence or co-roles of these pathogens in CD. Despite of the rationale why these pathogens were investigated individually for their role in CD, the outcome of each study may contributed to the controversy as to which microorganism really causes inflammation in CD, or which bacterium play a more important role in CD. For sure, it is challenging to use standard experimental techniques and less specialized skills to study diverse microorganisms together in individual clinical samples. This study is designed to address these challenges in an attempt to develop the necessary protocols to evaluate the role of co-multiple pathogens in gastrointestinal tract inflammation. Consequently, we focused on development of a multiplex PCR based on rapid DNA extraction, and multi-color fluorescent in situ hybridization (*m*-FISH) on based specific oligonucleotide sequences.

## Results

### Use of DNAzol^®^ for rapid extraction of DNA from diverse pathogens

**Fig. 1:**
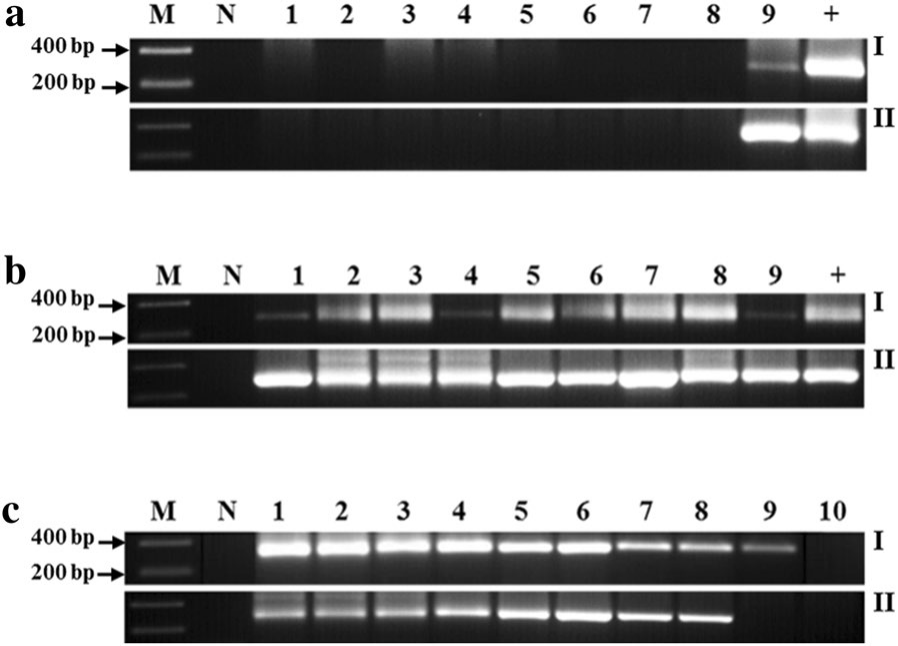
Specificity and sensitivity of the *IS900* nPCR in DNA extracts from the modified DNAzol^®^ and the standard phenol/chloroform/isoamyl-alcohol DNA extraction protocols. nPCR based on the *IS900* specific to MAP was performed on DNA template extracted by the standard phenol/chloroform/isoamyl-alcohol DNA extraction (I) and the modified DNAzol^®^ DNA extraction technique (II). A 298 bp fragment on 2% agarose gel is positive for MAP. **a** (1) Non-pathogenic *E. coli* strain K-12; (2) *S. aureus*; (3) *L. monocytogenes*; (4) *K. pneumoniae*; (5) *M. smegmatis*; (6) *M. avium* subspecies *avium*; (7) *M. xenopi*; (8) *M. fortuitum* subspecies *fortuitum*; (9) MAP Clinical Strain JF7. **b** (1) MAP Strain 1; (2) MAP Strain 3; (3) MAP Strain 8B; (4) MAP Para 18; (5) MAP UCF3; (6) MAP UCF5; (7) MAP UCF7; (8) MAP Linda; (9) MAP MS137. **c** Serial dilution of MAP UCF4 DNA concentrations were analyzed by nPCR. (1) 31.7 ng/μL; (2) 3.17 ng/μL; (3) 317 pg/μL; (4) 31.7 pg/μL; (5) 3.17 pg/μL; (6) 317 fg/μL; (7) 31.7 fg/μL; (8) 3.17 fg/μL; (9) 317 ag/μL; (10) 31.7 ag/μL. +: MAP UCF4; N: No DNA; M: molecular weight marker

Several attempts were performed to modify the DNAzol^®^ technique to extract genomic DNA from diverse microorganisms and to evaluate the sensitivity and specificity of the new protocol, which we compared to the standard phenol/chloroform/isoamyl-alcohol DNA extraction protocol. A 1 h-DNAzol^®^ protocol was developed. Following MAP-specific nPCR analysis, DNAzol^®^ extracts provided excellent PCR amplicons compared to the standard DNA extraction protocol. As shown in Fig. 1a, b, the oligonucleotide primers used in the *IS900* nPCR confirmed the specificity of the assay to MAP DNA. All non-MAP DNA extracts were negative for a 298 bp MAP amplicons. Moreover, the positive PCR DNA bands were more intense with better resolution when DNAzol^®^ extracts were used. Sensitivity of the *IS900* nPCR assay was evaluated against serial dilutions of DNA templates from the two DNA extractions techniques. When comparing the sensitivity of the *IS900* nPCR between the two DNA extraction techniques, the DNAzol^®^ extraction technique showed better sensitivity when examining serial dilutions of colony forming units (CFU) of MAP compared to the phenol/chloroform/isoamyl-alcohol DNA extraction technique. The minimum CFU detected by *IS900* nPCR after DNAzol^®^ DNA extraction was ~ 2 CFU compared to ~ 5 CFU when the phenol/chloroform/isoamyl-alcohol DNA extract was used. The sensitivity of the *IS900* nPCR was not significantly different when DNA extracts were used from both protocols (Fig. 1c). Specifically, the minimum amount of MAP genome detected by the *IS900* nPCR from DNA extracts from the two protocols ranged between ~ 3.17 fg/L to 317 ag/μL.

### Use of multiplex PCR on intestinal bacterial culture and tissue

**Fig. 2:**
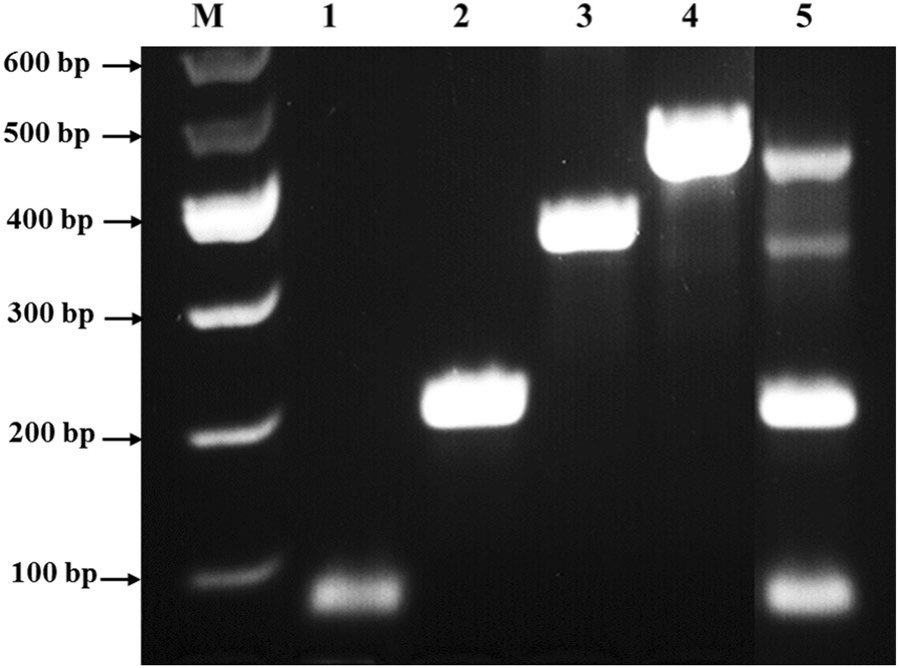
Validation of multiplex PCR using individual and combined oligonucleotide primer sets and DNA extracts from mixed bacterial culture. Multiplex PCR was performed on DNA extracts from mixed bacterial cultures. DNA template was extracted using the modified DNAzol^®^. (1) Non-pathogenic *E. coli* strain K-12 *18s* primers were used (171 bp); (2) MAP UCF4 *IS900* AV1/AV2 primers were used (298 bp); (3) *K. pneumoniae 23s* primers were used (493 bp); (4) *Mycobacterium avium* complex (MAC) *IS1311* primers were used (534 bp); (5) a cocktail of the 4 primer sets mentioned above were used. M: DNA molecular weight marker

We engineered an all in one multiplex PCR protocol that facilitates the detection of DNA from multiple bacteria in a single intestinal tissue biopsy. First, the test was validated against DNA from a mixture of bacterial culture, which included non-pathogenic *E. coli* strain K-12, *K. pneumoniae*, and MAP. As shown in Fig. 2, the oligonucleotides primers were specific to their corresponding microorganism. There was no cross amplification between any of the primers used and DNA from any of the other microorganisms. Specifically, no cross amplification was observed when individual primers set were used against DNA from mixed cultures and when mixture of the primers sets were used against individual bacterial DNA extracts. It is worth noting that we did not use DNA from AIEC strain LF82 due to laboratory biosafety level restrictions. In lieu of this, primers for AIEC strain LF82 were developed and tested against non-pathogenic *E. coli* strain K-12. The specificity of these primers were confirmed and validated against DNA and culture from non-pathogenic *E. coli* strain K-12. To add more support to the specificity of the MAP primers, *Mycobacterium avium* complex (MAC) primers were used to allow detection of non-MAP mycobacterial DNA in clinical samples, if present.

**Fig. 3:**
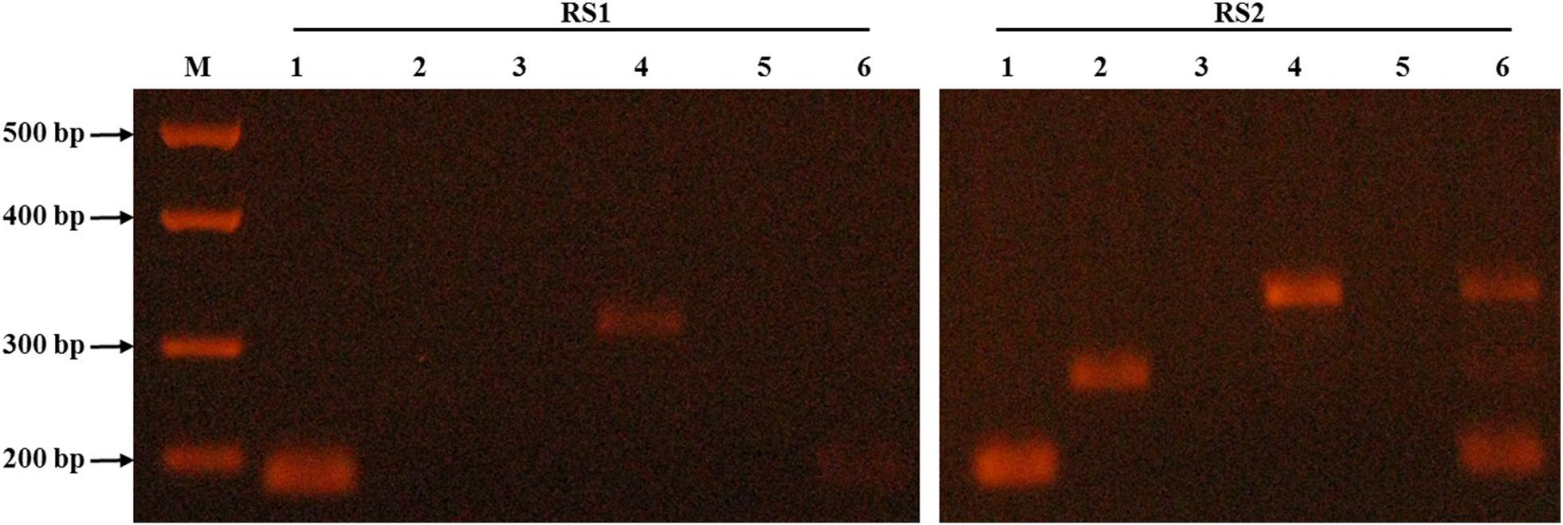
Validation of multiplex PCR using individual and combined oligonucleotide primer sets and DNA extracts from IBD tissue. Multiplex PCR was performed on DNA extracts from intestinal tissue samples. DNA template was extracted using the modified DNAzol^®^. RS1: ulcerative colitis (UC) patient; RS2: Crohn’s disease (CD) patient; (1) Non-pathogenic *E. coli* strain K-12 *18s* primers were used (171 bp); (2) MAP UCF4 *IS900* AV1/AV2 primers were used (298 bp); (3) AIEC strain LF82 g*ipA* primers were used (357 bp); (4) *K. pneumoniae 23s* primers were used (493 bp); (5) *Mycobacterium avium* complex (MAC) *IS1311* primers were used (534 bp); (6) a cocktail of the 5 primer sets mentioned above were used. M: DNA molecular weight marker

Following optimization of DNA templates, oligonucleotide primer and PCR temperature conditions, the modified DNAzol^®^ and the multiplex PCR protocols were used to evaluate intestinal biopsies from patients with UC (RS1) and CD (RS2). As shown in Table 1 and Fig. 3, DNA from both non-pathogenic *E. coli* strain K-12 (171 bp) and *K. pneumoniae* (493 bp) were detected in both RS1 and RS2 samples. MAP DNA (298 bp) was only detected in RS2 and not from RS1. AIEC strain LF82 (357 bp) was not detected in all samples.

**Table 1:** Multiplex PCR and *m*-FISH results for intestinal tissue from IBD

Microorganism	Intestinal tissue			
	RS1		RS2	
	Multiplex PCR	*m*-FISH	Multiplex PCR	*m*-FISH
*Non*-*pathogenic E. coli* strain K-12	+	+	+	+
*AIEC* strain *LF82*	−	−	−	−
*MAP*	−	−	+	+
*K. pneumoniae*	+	+	+	+
MAC	−	ND	−	ND

AIEC: adherent-invasive *Escherichia coli*; MAP: *Mycobacterium avium* subspecies *paratuberculosis*; MAC: *Mycobacterium avium* complex; *m*-FISH: multi-color fluorescent in situ hybridization; RS1: ulcerative colitis patient; RS2: Crohn’s disease patient; ND: not done

### Microscopic staining and *m*-FISH on intestinal bacterial culture and tissue

**Fig. 4:**
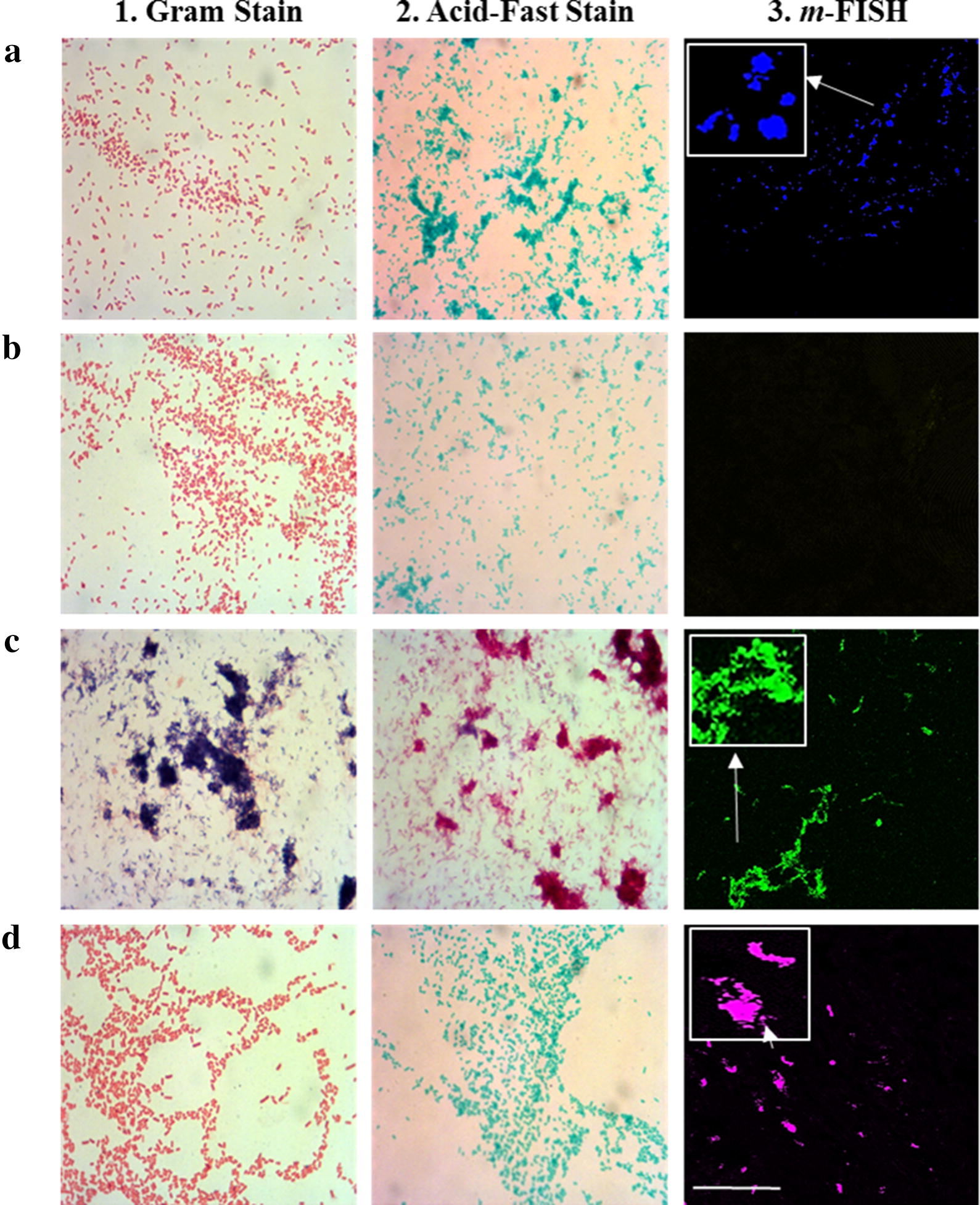
Gram stain, acid-fast stain and *m*-FISH detection of bacterial cultures. Gram stain (1), acid-fast stain (2), and oligonucleotide *m*-FISH (3) images of bacterial cultures: **a**, **b** Non-pathogenic *E. coli* strain K-12; **c** MAP UCF4; and **d** *K. pneumoniae*. For -*m*-FISH detection: **a**3 *m*-FISH using non-pathogenic *E. coli* K-12 *18s* probe labeled with AF647 fluorophore; **b**3 *m*-FISH using AIEC strain LF82 g*ipA* probe labeled with AF568 fluorophore; **c**3 *m*-FISH using MAP UCF4 *IS900* AV1 probe labeled with AF488 fluorophore; and **d**3 m-FISH using *K. pneumoniae 23s* probe labeled with AF546 fluorophore. All microscopic images were obtained at 1000 × magnification. White measurement bar found in D3 represents 100 μm. All *m*-FISH images were obtained using CSLM

Gram and acid-fast stains were used to verify the presence and identity of the microorganisms used in this study. As shown in Fig. 4, a 1000× amplification images of bacteria stained with Gram stains (Fig. 4-1) and acid-fast stains (Fig. 4-2) were illustrated. The *m*-FISH probes were specific to their corresponding microorganisms and there was no cross hybridization between probes used in this study (Fig. 4-3).

**Fig. 5:**
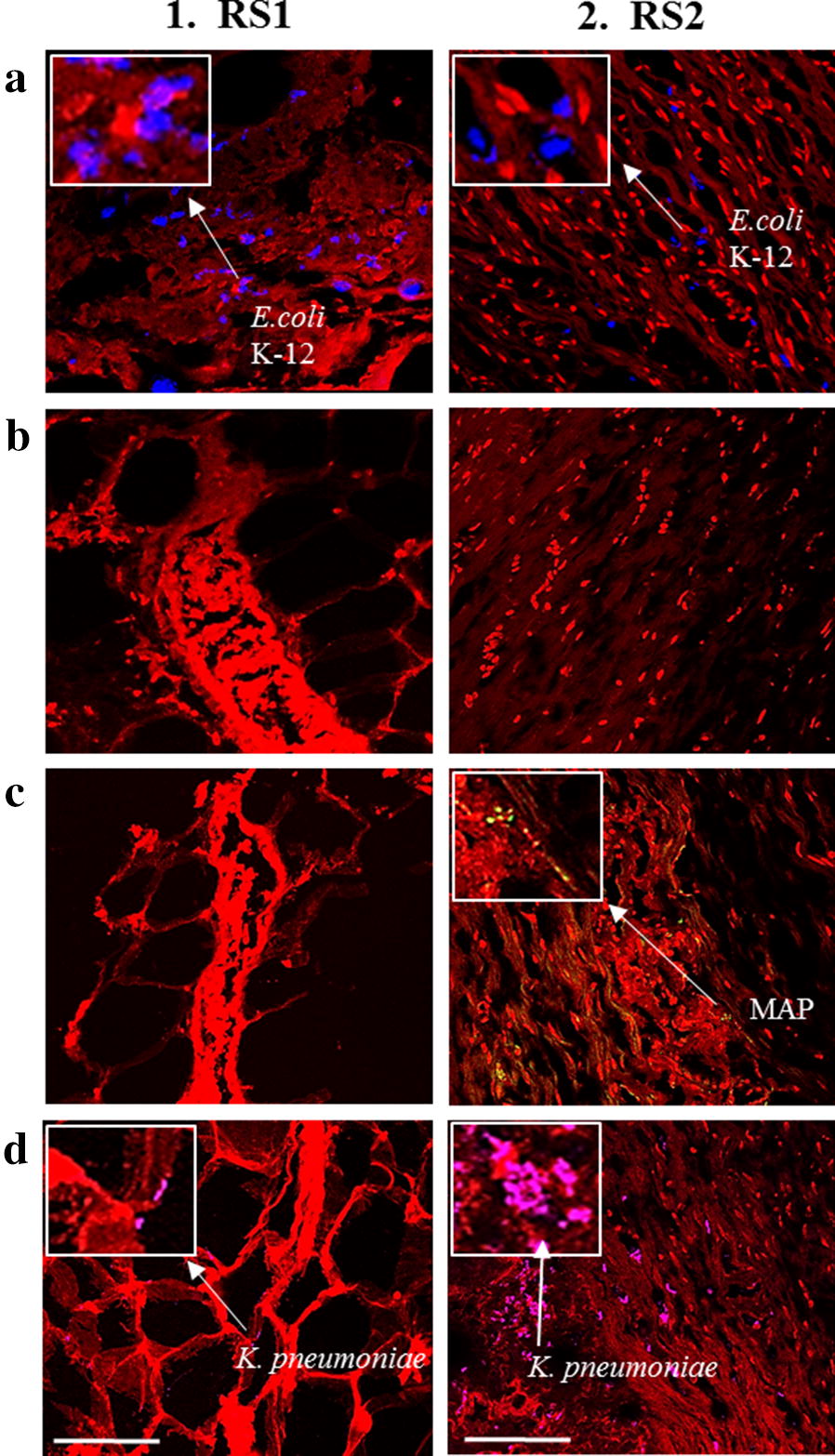
Use of *m*-FISH using individual probes to detect multiple pathogens in intestinal tissue. Oligonucleotide *m*-FISH analysis in tissue sections from patients with UC (RS1) and CD (RS2) was done targeting for: **a** Non-pathogenic *E. coli* strain K-12; **b** AIEC strain LF82; **c** MAP; and **d** *K. pneumoniae*. Images illustrating DAPI are in red. The individual *m*-FISH probes that were used were: **a** non-pathogenic *E. coli* strain K-12 *18s* probe labeled with AF647 fluorophore; **b** AIEC strain LF82 g*ipA* probe labeled with AF568 fluorophore; **c** MAP UCF4 *IS900* AV1 probe labeled with AF488 fluorophore; and **d** *K. pneumoniae 23s* probe labeled with AF546 fluorophore. White measurement bars found in D1 and D2 represents 20 μm. All *m*-FISH images were obtained using CSLM

For the IBD patient biopsy samples (RS1 and RS2), individual *m*-FISH probes were used along with DAPI (red fluorescence) to detect both the targeted bacteria and the tissue (Fig. 5). As with the multiplex PCR, *m*-FISH for non-pathogenic *E. coli* strain K-12 (AF647 fluorophore, blue fluorescence) and *K. pneumoniae* (AF546 fluorophore, magenta fluorescence) showed positive signaling in RS1 and RS2 tissue biopsies (Table 1, Fig. 5a-1, 2 and d-1, 2), while only MAP (AF488 fluorophore, green fluorescence) was detected in the RS2 sample (Table 1, Fig. 5c-1, 2). The AIEC strain LF82 (AF568 fluorophore, yellow fluorescence) was not detected in either RS1 or RS2 tissue biopsies (Table 1, Fig. 5b-1, 2).

### Use of multiple oligonucleotide probes via *m*-FISH in IBD tissue

**Fig. 6:**
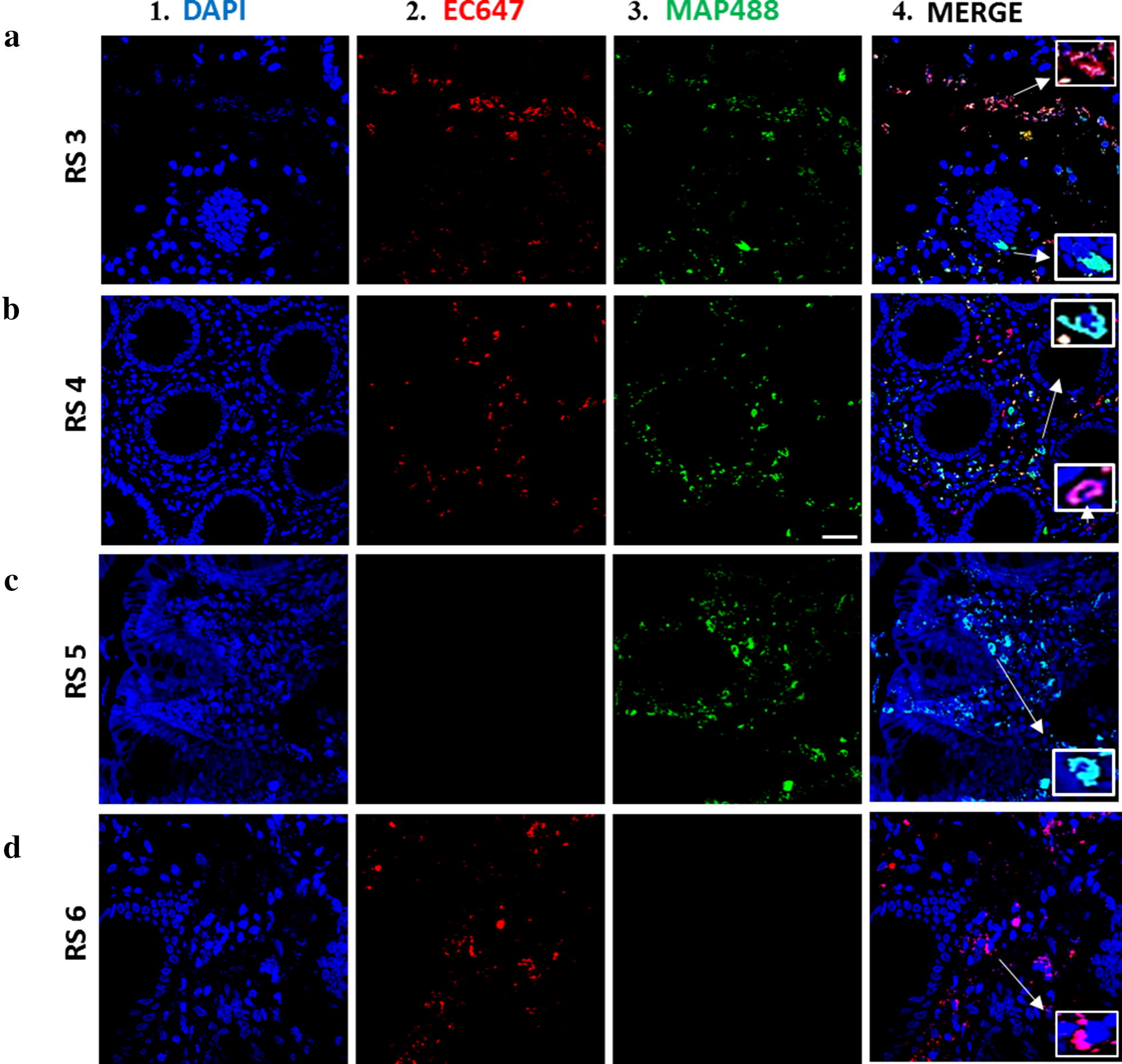
Use of *m*-FISH using combined probes to detect multiple pathogens in intestinal tissue from CD. Oligonucleotide *m*-FISH images of intestinal tissue sections from four CD patients (**a** RS3, **b** RS4, **c** RS5, and **d** RS6). A combined probes mixture including (2) EC647 (non-pathogenic *E. coli* strain K-12 *18s* probe labeled with AF647 fluorophore) and (3) MAP488 (MAP UCF4 *IS900* AV1 probe labeled with AF488 fluorophore) were used in all tissue sections. (1) DAPI staining and (4) merged images between DAPI and the corresponding probe. White measurement bar located in B3 represents 20 μm. All *m*-FISH images were obtained using CSLM

After optimization of the *m*-FISH protocol, we used two set of probes together to detect the presence or absence or co-presence of multiple microorganisms in same tissue sections. We tested tissue sections from four CD subjects (RS3, RS4, RS5, RS6) using our *m*-FISH. As shown in Fig. 6, images illustrate DAPI staining (blue fluorescence; a-1, b-1, c-1, d-1), non-pathogenic *E. coli* strain K-12 (AF657 fluorophore, red fluorescence, a-2, b-2, c-2, d-2), and MAP (AF488 fluorophore, green fluorescence, a-3, b-3, c-3, d-3). For RS3 and RS4, both non-pathogenic *E. coli* strain K-12 and MAP bacteria were successfully detected (Fig. 6a, b). RS5 (Fig. 6c-4) had only positive signaling for MAP, while RS6 (Fig. 6d-4) had only positive signaling for non-pathogenic *E. coli* strain K-12.

## Discussion

Recent studies have strongly supported the role of microbial infection in IBD development [7–12]. It is still unclear which pathogen is found more readily in either UC or CD patients, where recent studies are more focused on detecting one pathogen at a time [8, 10, 11, 18, 19]. In this study, the investigation of the presence of multiple pathogens including MAP, *E. coli* strains, and *K. pneumoniae*, was done to fully understand the role of possible co-infection or co-occurrence of multiple pathogens in individual IBD patients.

The development of a multiplex PCR protocol coupled with an *m*-FISH detection protocol was successful, which together can detect multiple bacterial pathogens in a single sample. Along with this, a newly modified DNA extraction protocol was used in order to process the samples faster (~ 1 h) than previously used techniques (2–3 days) [5, 6, 8]. Overall, combining all three protocols has produced a faster, more efficient way into detecting multiple bacterial species in one test sample.

The effectiveness of the two DNA extractions techniques that were used in this study was established, where the modified DNAzol^®^ technique showed similar specificity and sensitivity to the “traditional” phenol/chloroform/isoamyl-alcohol DNA extraction technique (Fig. 1). The modified DNAzol^®^ DNA extraction technique however has the advantage over the “traditional” DNA extraction due to the simplistic and the less time consuming protocol, thus could potentially lead to more samples being processed at a time. Also, due to the cost-effectiveness of the modified DNAzol^®^ DNA extraction protocol, which has a 1.5-fold lower cost than the “traditional” DNA extraction protocol, the modified protocol could be a better DNA extraction method to use in the clinical setting [20].

We developed a multiplex PCR that is specific to detect pathogens’ targets when DNA or bacteria were used purely or in a mixture (Figs. 2, 3). This technique was validated using DNA extracts from intestinal tissue from IBD subjects (Tables 1, 3). The labeling of oligonucleotide primers used in multiplex PCR with fluorophores in our *m*-FISH confirmed the identity of the microorganisms used in this study and the results obtained by multiplex PCR from intestinal tissue samples (Figs. 4, 5).

*m*-FISH illustrations validated the efficiency of the multiplex PCR in detecting multiple pathogens within hours using one protocol and limited reagents and steps. The significance of this technique will be tremendous for those investigating the role of multiple pathogens in tissue sections from patients with idiopathic or multifactorial diseases. Since CD is considered a syndrome with multifactorial etiology, the outcome of this study will provide new tools toward understanding which microorganism(s) is/are play significant role in disease pathogenies. As expected, both RS1 (UC) and RS2 (CD) were positive for non-pathogenic *E. coli* strain K-12 and *K. pneumoniae* (Fig. 6). However, detection of MAP in RS2 tissue (CD) and not in RS1 (UC) by multiplex PCR and validated with images by *m*-FISH support published reports of association of MAP with CD [5, 6, 8, 9, 13, 21]. Astonishing, both multiplex PCR and *m*-FISH did not detected the AIEC strain LF82 in the two IBD patients samples, where previous studies have shown that this bacterium has been associated with IBD pathogenesis [7, 10, 12, 18]. This could be due to the small sample size in this study, but also could be due to the possibility that the *gipA* gene being amplified in the AIEC strain LF82 could be a low copy gene target [22, 23]. The virulent gene *gipA* was chosen as the target gene for amplification in AIEC strain LF82 is due to its association with AIEC infection in Peyer’s patches of CD patients [22, 23].

When comparing the intensities of the multiplex PCR DNA bands and the *m*-FISH signaling of non-pathogenic *E. coli* strain K-12 with the signaling of *K. pneumoniae* in both patient samples, it is evident that the dysregulation of commensal *E. coli* could potentially play a role in a higher rate of *K. pneumoniae* infection in CD patients (Figs. 3, 5a, d). The data suggests that the more *K. pneumoniae* signaling is present in the CD patient (RS2) sample, the less non-pathogenic *E. coli* strain K-12 signaling is present. This could suggest and confirm that an opportunistic infection of *K. pneumoniae* can occur if there is a dysfunction of the microbiome in IBD patients due to the dysregulation of commensal bacteria, such as *E. coli* strain K-12 [11, 24–28]. With the increasing influx of *K. pneumoniae* in the CD patient more so in the UC patient, it could be a potential pathogen to investigate for CD pathogenesis studies.

The *m*-FISH assay on tissue from additional four other CD patients (RS3–RS6) showed that we able to detect and visualize multiple bacterial species in a single biopsy sample (Fig. 6). Further testing of more tissue from other diseases using the *m*-FISH probes and multiplex PCR for different microorganisms, such as other commensal bacteria species like *Bacteroidetes fragilis* and other opportunistic bacterial species like *Pseudomonas aeruginosa*, would be helpful to further validate these techniques to help elucidate idiopathic diseases with multiple etiology [29, 30].

Overall, this study was done as a pilot study in order to verify and examine these newly developed protocols, and as such, more IBD patient samples are required for future examination.

## Methods

### Bacterial cultures

A total of ten MAP strains, five other *Mycobacterium* species, and four non-*Mycobacterium* species were used in this study (Table 2). *Mycobacterium* species including MAP were cultured in BD Bactec™ MGIT™ Para-TB medium (Becton, Dickinson and Company©) tubes supplemented with 800 μL of Bactec™ MGIT™ Para-TB Supplement (Becton, Dickinson and Company©) and incubated at 37 °C until optimal growth was achieved. *E. coli*, *Staphylococcus aureus* and *K. pneumoniae* were cultured in Luria broth (LB broth, Fisher Scientific^®^) at 37 °C. *Listeria monocytogenes* was cultured in brain heart infusion broth (BHI broth, Fisher Scientific^®^) at 37 °C.

**Table 2:** Microorganisms used in this study

Microorganism	Source
MAP Strain 1	CD breast milk
MAP Strain 3	CD intestinal tissue
MAP Strain 8B	CD blood
MAP Para 18	ATCC 19698
MAP UCF3	CD intestinal tissue
MAP UCF4	CD intestinal tissue
MAP UCF5	CD intestinal tissue
MAP UCF7	CD intestinal tissue
MAP Linda	ATCC 43015
MAP MS137	CD intestinal tissue
*Mycobacterium smegmatis*	ATCC 27199
*Mycobacterium avium* subspecies *avium*	ATCC 25291
MAP clinical strain JF7	HIV blood
*Mycobacterium xenopi*	ATCC 19971
*Mycobacterium fortuitum* subspecies *fortuitum*	ATCC 23031
*Non*-*pathogenic Escherichia coli* Strain K-12	ATCC 8739
*Staphylococcus aureus*	ATCC 25932
*Klebsiella pneumoniae*	ATCC 13883
*Listeria monocytogenes*	ATCC 19112

MAP: *Mycobacterium avium* subspecies *paratuberculosis*

### Intestinal tissue

Intestinal tissue from one UC (RS1) and from five CD (RS2, RS3, RS4, RS5, RS6) patients were used in this study. Tissue samples used in this originated from subjects participated in earlier study and the unused tissue were stored at − 80 °C in Dr. Saleh A. Naser’s laboratory. These tissue were obtained following the University of Central Florida Institutional Review Board #IRB00001138 approval. Although limited information is available about these tissue samples, the tissue were useful for this study since the intention is limited to validation of detection of selected microorganisms in IBD gut.

### DNA extraction

#### Preparation of cell pellets/intestinal tissue lysate for DNA extraction

A volume of 1 mL of bacterial culture in 1.5 mL microcentrifuge tube was centrifuged at 13,000 RPMs for 2.5 min at room temperature. Supernatant was discarded and bacterial cell pellet was re-suspended in 500 μL of tris–EDTA buffer (TE buffer). For intestinal tissue, approximately 1 g tissue block was placed in tissue grinder (Precision™) with 1 mL of saline solution and was homogenized for 15 min. The tissue homogenate was then added to a lysing matrix B tubes (MP Biomedicals©) and was subjected to sonication using FastPrep FP120 Cell Disrupter at 6.0 m/s for 30 s in a (Thermo Savant™). The lysate was then centrifuged at 13,000 RPMs for 20 min. The supernatant was then removed from each tube and stored at − 20 °C until further use.

DNA extractions of both bacterial cell pellets and intestinal tissue lysate were performed following our modified DNAzol^®^ (ThermoFisher Scientific^®^) DNA extraction protocol and our traditional phenol/chloroform/isoamyl DNA extraction method as described previously [5, 6, 8].

#### Modified DNAzol^®^ DNA extraction protocol

Each tube containing bacterial culture pellet or intestinal tissue lysate in 500 μL of TE was subjected to DNA extraction by a protocol that utilizes DNAzol^®^ as previously described [5, 6]. Briefly, a total of 1.0 mL of DNAzol^®^ was added to bacterial culture pellets or intestinal tissue lysates suspended in 500 μL TE. After mixing, a 400 μL of 100% isopropanol was added to each tube and then incubated for 15 min at room temperature. Following centrifugation at 8000 RPMs for 6 min, the supernatant was discarded and DNA pellets were then washed with 500 μL of DNAzol^®^ at 8000 RPMs for 5 min. DNA pellets were then washed again in 1.0 mL of 75% ethanol at 8000 RPMs for 5 min and then dried via a speedvac for 5 min. Dried DNA pellets were then dissolved in 50 μL of TE buffer and stored at − 20 °C until further use.

#### Phenol/chloroform/isoamyl DNA extraction protocol

Each tube containing bacterial culture pellet or tissue lysate in 500 μL of TE was subjected to DNA extraction by a protocol that utilizes phenol/chloroform/isoamyl alcohol as previously described [8]. Briefly, tubes were incubated in a heat block for 30 min at 100 °C and then placed on ice for 15 min. Tubes were then centrifuged at 12,000 RPMs at 4 °C for 10 min. Supernatants were transferred into 2.0 mL Phase Lock Gel™ tubes (Fisher Scientific^®^) and then mixed with 200 μL of phenol/chloroform/isoamyl-alcohol (Fisher Scientific^®^). Tubes were centrifuged at 12,000 RPMs at 4 °C for 5 min, where supernatants transferred into new 1.5 mL microcentrifuge tubes containing 100% chilled ethanol and stored at − 20 °C overnight. Next day, tubes are thawed and centrifuged at 12,000 RPMs at 4 °C for 10 min and the supernatants discarded. DNA pellets were washed with 80% chilled ethanol, dried in a speedvac for 15 min and re-suspended in 50 μL TE buffer for storage at − 20 °C until further use.

### Validation of DNAzol^®^ extraction method by MAP *IS900* nPCR

MAP-specific nPCR based on *IS900* derived oligonucleotide primers (Table 3) was used to evaluate the efficiency of the modified DNAzol^®^ protocol compared to the phenol/chloroform/isoamyl alcohol protocol. In the first round, the PCR reaction consisted of 25 μL master mix (2× solution containing *Taq* DNA polymerase, dNTPs, MgCl_2_ and reaction buffers, Promega©), 5 μL betaine (Sigma-Aldrich©), 1 μL of P90 and 1 μL of P91 oligonucleotide primers, 8 μL of Millipore H_2_O, and 10 μL of DNA. The PCR cycling conditions were: 95 °C for 5 min; 35 cycles of 95 °C for 60 s, 58 °C for 90 s, and 72 °C for 90 s; and a final extension of 72 °C for 10 min. For the second round of PCR, the same reagents were used from the first round with 5 μL of P90/P91 product and AV1/AV2 oligonucleotide primers. The PCR cycling conditions were: 95 °C for 5 min; 35 cycles of 95 °C for 30 s, 60 °C for 30 s, and 72 °C for 60 s; and a final extension of 72 °C for 10 min. Amplified DNA was then analyzed on a 2% agarose gel, and a 298 bp band was considered positive for MAP. DNA from MAP clinical strain UCF4 was used a positive control. The negative control consisted of all reagents except DNA.

**Table 3:** Nucleotide primers used in multiplex PCR and *m*-FISH probes

Bacterial gene target	nPCR primers	Multiplex PCR primers	*m*-FISH probe
*Mycobacterium avium* subspecies *paratuberculosis* (MAP) *IS900*	P90: 5′-GTTCGGGGCCGTCGCTTAGG-3′ (BLAST E-value: 2e−04) P91: 5′-GAGGTCGATCGCCCACGTGA-3′ (BLAST E-value: 2e−04) AV1: 5′-ATGTGGTTGCTGTGTTGGATGG-3′ (BLAST E-value: 1e−05) AV2: 5′-CCGCCGCAATCAACTCCAG-3′ (BLAST E-value: 5e−04)	AV1: 5′-ATGTGGTTGCTGTGTTGGATGG-3′ (BLAST E-value: 1e−05) AV2: 5′-CCGCCGCAATCAACTCCAG-3′ (BLAST E-value: 5e−04)	AV1: 5′-AF488 ATGTGGTTGCTGTGTTGGATGG-3′ (BLAST E-value: 1e−05)
*Mycobacterium avium* complex (MAC) *IS1311*	NA	Forward: 5′-AAACGACCAAGGATCACTACCGAG-3′ (BLAST E-value: 1e−06) Reverse: 5′-GTCGAGGAACACATACGGGAAGT-3′ (BLAST E-value: 4e−06)	NA
Non-pathogenic *E. coli* strain K-12 *18s*	NA	Forward: 5′-CCGCATAACGTCGCAAGACC-3′ (BLAST E-value: 6e−04) Reverse: 5′-CGTAGGAGTCTGGACCGTGTC-3′ (BLAST E-value: 2e−04)	5′-AF647 GGTCTTGCGACGTTATGCGG-3′ (BLAST E-value: 6e−04)
AIEC strain LF82 *gipA*	NA	Forward: 5′-GCTGTGTGCGCTTCGTCTAC-3′ (BLAST E-value: 4e−08) Reverse: 5′-GATGGTAATTCTCGACTCCAGCGA-3′ (BLAST E-value: 2e−07)	5′-AF568 GTAGACGAAGCGCACACAGC-3′ (BLAST E-value: 4e−08)
*K. pneumoniae* *23* *s*	NA	Forward: 5′-TGGCAGTCAGAGGCGATGAAG-3′ (BLAST E-value: 1e−04) Reverse: 5′-CTTTCCCTCACGGTACTGGTTCA-3′ (BLAST E-value: 0.002)	5′-AF546 CTTCATCGCCTCTGACTGCCA-3′ (BLAST E-value: 0.001)

BLAST E-value: basic local alignment search tool expected value

### Development of multiplex PCR

All oligonucleotide primers were designed and then purchased from Eurofins Genomics© (Table 3). Briefly, 10 μL of DNA containing 17 ng/μL of bacterial DNA or 50 ng/μL tissue DNA were added into a 200 μL-microcentrifuge tube containing 25 μL of PCR Master Mix (2× solution containing *Taq* DNA polymerase, dNTPs, MgCl2 and reaction buffers, Promega©), 5 μL of betaine (Sigma-Aldrich©), and 1 μL of each oligonucleotide primer (10 μM forward and 10 μM reverse primer for each bacterial species (MAP, MAC, non-pathogenic *E. coli* strain K-12, AIEC strain LF82, and *K. pneumoniae*). The PCR cycling conditions were: 95 °C for 5 min; 35 cycles of 95 °C for 30 s, 60 °C for 30 s, and 72 °C for 60 s; and a final extension of 72 °C for 10 min. The products of the multiplex PCR were analyzed on a 3% agarose gel, where the following base pairs bands are considered positive for the bacterial species tested: 171 bp (non-pathogenic *E. coli* strain K-12), 298 bp (MAP), 357 bp (AIEC strain LF82), 493 bp (*K. pneumoniae*), and 543 bp (MAC). For the bacterial DNA positive controls, all tested bacterial species were first separated into individual tubes and underwent DNA extraction/multiplex PCR. Once successful multiplex PCR was done on the bacterial DNA positive controls individually, all tested bacterial species were then added into one tube and then underwent DNA extraction/multiplex PCR. The negative control for the multiplex PCR step that was used had all of the necessary PCR reagents except for the DNA.

### Development of multi-color fluorescent in situ hybridization (*m*-FISH) for imaging for gut bacteria

#### Preparation of bacterial slides for m-FISH

A volume of 1 mL of bacterial culture in 1.5 mL microcentrifuge tube was centrifuged at 13,000 RPMs for 2.5 min at room temperature. The supernatant was removed and the culture pellet was washed with 500 μL of TE and then centrifuged again at 13,000 RPMs for 2.5 min at room temperature. After centrifugation, the supernatant was removed and the culture pellet was re-suspended in 100 μL of TE. Of which, 20 μL suspension was placed on each slide, air dried and then heat fixed. Slides were then incubated in 4% paraformaldehyde (PFA, Fisher Scientific^®^) overnight at 4 °C on a shaker at 50 rpm.

#### Preparation of intestinal tissue slides for m-FISH

Intestinal tissue sections were obtained using previously established protocols [31, 32]. In brief, PFA fixed tissue specimens (1 g each) were placed in perforated cassettes and immersed in ascending concentrations of ethanol (70%, 90%, and 100%) on a Leica processing system (TP 1020) for dehydration followed by clearing in (Xylene 50:Ethanol 50), and 100% Xylene solutions. Next, intestinal tissue was embedded in melted paraffin (60 °C) and allowed to solidify to 4 °C. Solidified blocks were then cut using tissue microtome (HM 325 Microm; Medical Equipment Source) into consistent 5 μm thickness serial tissue sections and placed on Colorfrost Plus microscope slides (Fisher Scientific^®^). Before histology evaluation, sections were put in 60 °C incubator for 30 min, and immersed into in xylene solution (Sigma-Aldrich©) for 10 min to remove extra wax and three separate washes with 100% ethanol, 95% ethanol, and 70% ethanol for 10 min each to rehydrate the tissue section. The tissue slides were air-dried after removal of paraffin, and incubated overnight at 4 °C with 4% PFA (Fisher Scientific^®^) on a shaker.

#### Labeling of probes with fluorophores

An *m*-FISH protocol was developed in order to visually allow the detection of multiple microorganisms in a single tissue section from intestinal biopsy sample. Oligonucleotide primers used earlier in multiplex PCR (Table 3) were labeled with fluorophores (Eurofins Genomics©) as follows: non-pathogenic *E. coli* strain K-12 *18s* (AF647 fluorophore, blue fluorescence), AIEC strain LF82 *gipA* (AF568 fluorophore, yellow fluorescence), MAP *IS900* (AV1 primer, AF488 fluorophore, green fluorescence), and *K. pneumoniae 23s* (AF546 fluorophore, magenta fluorescence) (Fig. 4). All *m*-FISH oligonucleotide probes were prepared by diluting probes to 1 μg/μL in TE buffer.

#### Gram and acid-fast staining

For confirmation of bacterial presence and identity, we used standard staining including Gram and acid-fast staining [33, 34]. To prepare the bacteria culture slides, a volume of 1 mL of bacterial culture in 1.5 mL microcentrifuge tube was centrifuged at 13,000 RPMs for 2.5 min at room temperature. The supernatant was removed and the culture pellet was washed with 500 μL of TE and then centrifuged again at 13,000 RPMs for 2.5 min at room temperature. After centrifugation, the supernatant was removed and the culture pellet was re-suspended in 100 μL of TE. Of which, 20 μL suspension was placed on a microscope slide, air dried and then heat fixed. Gram staining and acid-fast staining was done using the Fisher Scientific^®^ Gram staining kits and using their protocols.

#### Bacterial m-FISH on bacterial slides and IBD patient’s biopsy slides

After fixation with 4% PFA, the slides were then washed three separate times in 1× phosphate buffer saline (PBS) for 10 min each on a shaker. Next, a solution comprising of 100 μL of 1% sodium dodecyl sulfate (SDS) with 2 μL of 20 mg/mL of Proteinase K (Thermo Scientific™) was added directly to the slides in hybridization chambers (Corning^®^). The slides in the hybridization chambers were then incubated at 55 °C for 30 min. Inactivation of the Proteinase K was done by adding 200 μL of 0.2% glycine (Sigma-Aldrich©) to each slide and incubated for 3 min on a shaker. After inactivation, the slides were then washed three separate times in 1× PBS for 5 min each on a shaker. The slides were washed again in the following solutions for 1 min each on a shaker: 50% ethanol, 80% ethanol, 100% ethanol and then xylene. Next, the slides were washed once again with the following solutions for 1 min each on a shaker: 100% ethanol, 80% ethanol, and 50% ethanol. After the ethanol/xylene washes, the slides are then incubated in 1× PBS for 60 min on a shaker. The slides were then incubated in a pre-hybridization solution consisting of 2× saline-sodium citrate (SSC, Sigma-Aldrich©), 20% dextran sulfate (Fisher Scientific^®^), 50% formamide (Sigma-Aldrich©), 50 mM NaH_2_PO_4_ (Sigma-Aldrich©), and 1 mM EDTA (Fisher Scientific^®^) for 10 min at 50 °C. After incubation in the pre-hybridization solution, the slides are then placed in hybridization chambers and 20 μL of hybridization solution (pre-hybridization solution without the 20% dextran sulfate) was added directly to the samples. The hybridization solution also included 3 μL of the 1 μg/μL oligonucleotide fluorescent probe per slide for the bacterial species being detected. After the hybridization solution was added to the slides in the hybridization chambers, the slides were then incubated for 60 °C for 60 min and then 37 °C overnight. After incubation overnight, the slides were then washed with the following solutions for 15 min each on a shaker: 2× SSC, 1× SSC, 0.3× SSC in 40 °C water bath, and 0.3× SSC in room temperature in the dark. The slides were then washed three separate times with H_2_O for 15 min each on a shaker and then air-dried in the dark. A solution of DAPI/mounting medium (Vectashield^®^) was added to the slides and were sealed with a slide cover. The slides were analyzed using confocal scanning laser microscopy (CSLM). The images created were analyzed on ImageJ (National Institute of Health).

## Abbreviations

IBD: inflammatory bowel disease
UC: ulcerative colitis
CD: Crohn’s disease
MAP: *Mycobacterium avium* subspecies *paratuberculosis*
AIEC: adherent-invasive *Escherichia coli*
PCR: polymerase chain reaction
*m*-FISH: multi-color fluorescent in situ hybridization
CSLM: confocal scanning laser microscopy
nPCR: nested polymerase chain reaction
CFU: colony forming units
MAC: *Mycobacterium avium* complex
